# Variability in DNA Repair Capacity Levels among Molecular Breast Cancer Subtypes: Triple Negative Breast Cancer Shows Lowest Repair

**DOI:** 10.3390/ijms18071505

**Published:** 2017-07-12

**Authors:** Jaime Matta, Carmen Ortiz, Jarline Encarnación, Julie Dutil, Erick Suárez

**Affiliations:** 1 Department of Basic Sciences, Divisions of Pharmacology, Toxicology, Biochemistry and Cancer Biology, Ponce Health Sciences University—School of Medicine, Ponce Research Institute, Ponce 00716-2348, Puerto Rico; carmenortiz@psm.edu (C.O.); jencarnacion@psm.edu (J.E.); jdutil@psm.edu (J.D.); 2 Department of Biostatistics and Epidemiology, Graduate School of Public Health, University of Puerto Rico, Medical Sciences Campus, San Juan 00936-5067, Puerto Rico; erick.suarez@upr.edu

**Keywords:** breast cancer, molecular subtypes, phenotypic variability, DNA repair capacity, multinomial regression analysis, precision medicine

## Abstract

Breast cancer (BC) is a heterogeneous disease which many studies have classified in at least four molecular subtypes: Luminal A, Luminal B, HER2-Enriched, and Basal-like (including triple-negative breast cancer, TNBC). These subtypes provide information to stratify patients for better prognostic predictions and treatment selection. Individuals vary in their sensitivities to carcinogens due to differences in their DNA repair capacity (DRC) levels. Although our previous case-control study established low DRC (in terms of NER pathway) as a BC risk factor, we aim to study this effect among the molecular subtypes. Therefore, the objectives of this study include investigating whether DRC varies among molecular subtypes and testing any association regarding DRC. This study comprised 267 recently diagnosed women with BC (cases) and 682 without BC (controls). Our results show a substantial variability in DRC among the molecular subtypes, with TNBC cases (*n* = 47) having the lowest DRC (*p*-value < 0.05). Almost 80 percent of BC cases had a DRC below the median (4.3%). Low DRC was strongly associated with the TNBC subtype (OR 7.2; 95% CI 3.3, 15.7). In conclusion, our study provides the first report on the variability among the molecular subtypes and provides a hypothesis based on DRC levels for the poor prognosis of TNBC.

## 1. Introduction

Worldwide, breast cancer (BC) is the most common cancer affecting women [1]. In the U.S. and Puerto Rico, BC now accounts for 30% of all new cancers in women [2,3]. Molecular studies of BC have revealed distinct disease subtypes, each associated with different risk factors, etiology, incidence, prognosis, survival rates, treatment approaches, and responses [4,5,6,7]. While high-throughput gene expression analysis continues to reveal more distinctions between BC subtypes, their classification is still in flux. For example, what collectively has been known as triple-negative breast cancer (TNBC) is now yielding distinct genetic profiles for basal-like and claudin-low BC (the latter seeming to be an intermediate between basal-like and luminal BC) [8,9]. Despite gene expression profiling’s ability to supply vital genetic information for stratifying patients [10], it is costly, not uniformly available, and the subset of genes analyzed vary by manufacturer. Therefore, immunohistochemistry (IHC) for receptor status and gene expression is a frequently used surrogate tool to classify BC subtypes. Under IHC auspices, Luminal A is ER+, PR+/−, HER2−; Luminal B is also positive for ER and PR+/− but is HER2+. HER2-enriched BC is ER−, PR−, HER2+. Triple-negative BC is negative for all three receptors and has the worst prognosis and treatment response [11,12].

For over a decade, our laboratory has focused, using lymphocytes as surrogate markers, on the role of DNA repair capacity (DRC) as risk factor for BC in women. We have previously shown in our large BC cohort (*n* = 824) that women with BC (*n* = 285) had a mean DRC of 2.40% (range: 0.14–15.00%); whereas the women without BC had a mean DRC level of 6.13% (range: 0.14–19.00%) [13]. In addition, we showed that the likelihood of developing BC increases by 64% for every 1% decrease in DRC measurement [13]. Therefore, our findings support what have been previously published, that low DRC is a marker that correlates with higher cancer risk [14,15,16]. In BC, the nucleotide excision repair (NER) pathway is particularly affected. NER deficiency is now well established as a DNA repair phenotype in BC, which suggests that it contributes to the etiology of both familial and sporadic BCs [13,16,17]. We also recently showed that ER status is associated with that defective repair phenotype [18]. This current study investigates whether DRC levels vary among BC subtypes and whether that variability is associated with an accompanying variance in risk of developing sporadic BCs.

## 2. Results

In the analysis of results, first an initial description of the study group was performed. Afterwards, a bivariate analysis was performed for assessing the association between different BC risk factors and molecular subtype of BC, in order to identify the potential confounders for this association. Finally, the magnitude of the association between BC molecular subtype and DRC was estimated, controlling for different confounding variables using a multinomial logistic regression model.

### 2.1. Description of Study Group

This study comprised 682 controls (women without BC) and 267 treatment-naïve women with recently diagnosed BC: 157 had Luminal A, 41 Luminal B, 22 HER2-positive, and 47 had triple-negative BC (TNBC). At the time of initial diagnosis, the age (years) distribution varied significantly (*p*-value < 0.05) by subtype; TNBC were the oldest (median 60 years), followed by Luminal A (median 58 years) and Luminal B (median 55 years) (Table 1). This distribution of BC molecular subtypes is consistent with national U.S. statistics [6,7,19,20,21].

### 2.2. Differential Distribution of DRC

**Figure 1:**
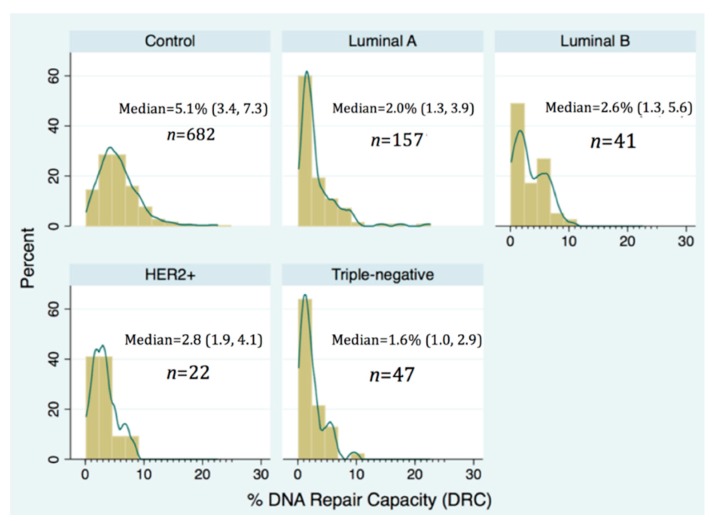
Distribution of DNA repair capacity (DRC) in controls and breast cancer cases stratified using the four principal molecular subtypes of breast cancer. The numbers included on each panel for each group include the median.

The DNA repair capacity (DRC in %) distribution by molecular subtype of BC showed a positive skew (were highly concentrated at low values) for Luminal A (median 2.0%; p25 = 1.3, p75 = 7.3) and TNBC (median 1.6%; p25 = 1.0, p75 = 2.9). After sorting the DRC (%), the value of the DRC (%) that reached 25% of the women (percentile 25) is different for each subtype: 3.4% for controls, 1.3% for Luminal A, 1.3% for Luminal B, 1.9% for HER2+ and 1.0% for TNBC. These differences were statistically significant (*p*-value < 0.05) (Table 1). In contrast, the DRC % distribution among controls was quite symmetrical (median 5.1%; p25 = 3.4, p75 = 7.3); however, this group showed the largest interquartile range (3.9) (Figure 1).

### 2.3. Molecular Subtypes of Breast Cancer by Different Characteristics

The bivariate analysis showed that the following factors had a significant association (*p*-value < 0.05) with molecular subtype: DRC, age, menopausal status, alcohol intake, vitamin intake, and HRT. Approximately 60% of the controls had DRC values above 4.3%, while only 17% of the women with TNBC women had DRC values above this median value. Among women with Luminal B tumors, only 19.5% reported having a family history of BC, while this distribution in controls was 33%. Around 72% of the women with tumors classified as TNBC were menopausal. Alcohol intake was more frequent in HER2+ (22.7%) followed by women in the control group (17.4%). Approximately 51% of women in the control group had multivitamin consumption (including calcium) and only 21.3% of the TNBC women reported this consumption. Among the post-menopausal women, 51.7% of the controls reported having taken HRT, while only 32.4% of the women with TNBC reported having undergone HRT therapy (Table 1).

### 2.4. Pathological Characteristics of Breast Tumors

Among women with BC, the results did not show significant differences (*p*-value > 0.05) regarding the type of BC (in situ, invasive, and mixed invasive) and the molecular subtypes. On the contrary, the grade of the tumor showed different patterns (*p*-value < 0.001) according to the molecular subtype of BC. Grade II cancer was the most prevalent among Luminal A and B subtypes; however, Grade III was the most prevalent in HER2+ and TN BCs (*p*-value < 0.001). Consistent with other findings [22,23], the highest prevalence of Grade III (most aggressive) BC occurred in the TNBC patients (30 of 44; 68%) (Table 2).

### 2.5. Magnitude of the Association

When ranked proportionally, more women with TNBC had a low DRC than any other BC subtype. The proportions of cases with low DRC were: 83% TNBC (39/47), 78% Luminal A (122/157), 77% HER2+ (17/22), 63% Luminal B (26/41). Low DRC was most strongly associated with women who had TNBC (adjusted OR: 7.2; 95% CI 3.3, 15.7). Women with HER2+ and Luminal A BC had virtually the same high association with low DRC (point estimates of the adjusted OR were 5.2 and 5.4, respectively). Women with Luminal B breast cancer had the weakest association between BC subtype and DRC levels (adjusted OR: 2.5; 95% CI 1.3, 4.9) and the least number of cases with a low DRC (Table 3).

## 3. Discussion

This study represents the first report that a low DRC (in relation to controls) is present in the four principal molecular BC subtypes and that is more pronounced in TNBC. Since our study is based on a large sample size, it suggests that significant phenotypic variability in terms of DRC exists amongst the four molecular subtypes studied. Our previous studies [13,16,18] and the study of Latimer et al. (2010) [17] had clearly established the critical importance of low DRC (measured in terms of NER pathway) as a risk factor for BC. Although the focus of our previous work was to study the relationship between DRC and BC, considering the disease as a single entity, we now aim to study this effect in terms of molecular subtypes.

The lowest DRC was associated with TNBC, the molecular subtype associated with the worst prognosis [22,24]. Among the molecular subtypes included, the highest adjusted OR (7.2) was found for TNBC. Our findings show that recently diagnosed, untreated women with TNBC, had a significantly lower DRC when compared to controls and women with Luminal A, Luminal B, and HER2+ BC. This may provide a hypothesis to at least partially explain why TNBC have a poorer prognosis when compared with other molecular subtypes. Our phenotypic measurement of DRC levels obtained from untreated women with BC confirms what is known about the prognosis of TNBC.

Our results obtained using a phenotypic assay to assess DRC are consonant with the findings reported by Ribeiro et al. (2013) at the gene expression level. This group found significant downregulation of 13 DNA repair genes, including five genes from the NER pathway (*ERCC1*, *XPA*, *XPD*, *XPG*, *XPF*) in TNBC [25]. Gene expression patterns were obtained following an RNA extraction from formalin-fixed paraffin embedded samples from 70 Luminal A, BC tumors and 80 TNBC tumors obtained from 150 women with BC in Italy. In addition to this group, Alexander et al. (2010) were able to establish prognostic markers for time to recurrence in TNBC through immunohistochemical assessment of key proteins in multiple DNA repair pathways. Among the four markers, a low expression of the NER protein XPF was associated with shorter time to recurrence. Moreover, this group developed a four-antibody model that was able to successfully identify high- and low-risk groups in terms of time to recurrence in TNBC [26]. These two studies, along with our findings, highlight the important role of NER in the biology of TNBC.

Given the research impetus in recent years for a more advanced precision medicine approach, the inclusion of phenotypic variability in DRC levels provides a tool for implementing that goal in BC diagnostic and treatment. Although the main therapeutic focus of DNA repair appears to be in the area of PARP inhibitors, DRC levels might allow us to distinguish subtle differences in BC molecular subtypes and prognosis. Since TNBC patients usually have the worst prognosis and acquire drug resistance more frequently than any other molecular subtype [27], our approach of using lymphocytes as surrogate markers of overall DRC could aid in the prediction of overall therapy response of women with TNBC. A new focus area in our studies is applying phenotypic measurement of DRC levels to study recurrence and metastasis in the large cohort of women with BC that we have studied for the last decade. Our findings may also prove useful in predicting (in terms of DRC levels) which molecular subtypes have a higher risk of recurrence and/or metastasis of BC, an important area in precision medicine.

## 4. Materials and Methods

### 4.1. Patient Recruitment

Patients were selected from our larger BC study (1183 patients and controls recruited 2006–2013). This study’s cohort comprised 949 Puerto Rican women age 21 or older: 267 with newly diagnosed BC (cases) and 682 without BC (controls). From a previous study [13] in which we recruited 824 Puerto Rican women, age 21 or older (285 newly diagnosed cases and 539 controls) power and sample size calculations were made. Sample size calculations performed initially revealed that a sample size of 824 participants (312 women with BC, 515 women without BC) would allow us to have a statistically significant odds ratio as low as 1.7 when the percent exposed to a low DRC among controls is 15% or higher (e.g., 15% controls are 21 to 30 years of age) with 5% significant level and 80% of statistical power. Selection bias was minimized by recruiting women who were getting routine gynecological screenings in the same clinics and hospitals where they would be treated if they were to develop BC. Those facilities represented 83% of the municipalities (65/78 counties) on the island.

To reduce the likelihood of including undiagnosed BC cases in our controls, only women who had normal results from a clinical breast exam and mammogram within the past six months were included. BC cases were limited to only recently diagnosed, histopathologically confirmed, treatment-naïve BC patients with primary tumors and pathology reports that included hormone receptor information. Because blood transfusions, radio- and chemotherapy can significantly affect DRC [28,29,30], patients who had received any of those treatments in the past five years were excluded from the study. Also excluded from this analysis were those with metastatic BC, secondary BC, breast metastases from another type of cancer, or any acquired or genetic immunodeficiency.

### 4.2. Use of Human Subjects

The Ponce Health Sciences University Institutional Review Board approved this study (IRB #130207-JM; Date: 13 February 2013). Each participant signed an informed consent form, giving us permission to draw their blood and review their pathology reports. All participants also completed an epidemiological questionnaire.

### 4.3. Blood Collection and Isolation of Lymphocytes

With the participants’ permission, we drew approximately 30 mL of peripheral blood into heparinized tubes and isolated the lymphocytes using the Ficoll gradient technique. Blood collection was performed during morning hours. Lymphocytes were suspended in 2 mL of freezing media (10% dimethyl sulfoxide, 39% RPMI 1640 medium, 50% fetal bovine serum, 1% antibiotic/antimycotic). Aliquots were stored in a −80 °C freezer for 1–3 weeks until thawed in batches for host-cell reactivation (HCR) assays.

### 4.4. DNA Repair Capacity Measurements

The isolated lymphocytes were used as surrogate markers of the patients’ overall DRC [31,32]. The cells were purified and grown, then the HCR assay was performed on them to measure in vivo DRC, as described in previous studies [13,16,33,34,35]. Briefly, the lymphocytes were transfected with a plasmid containing the luciferase reporter gene. Plasmids had been damaged with UVC prior to transfection. The cells’ ability to repair the foreign DNA was measured via HCR [35] within a specific time frame (40 h) that mirrored the true cellular process [32]. Results reflected the cells’ inherent DRC, measured primarily in terms of their NER pathway activity. Details about HCR’s sensitivity, specificity, and plasmid transfection efficiency have been published previously [13].

To calculate DRC, the luciferase activity after repair of the UVC-damaged plasmid DNA was compared with the undamaged plasmid DNA. The amount of residual luciferase remaining after the allotted repair time (activity in luminescence units) was a percentage that represented the amount of the individuals’ DRC. Because DRC is traditionally low in BC cases, results were analyzed in tertiles, as described in our previous study [13]. However, to perform the proposed statistical analyses, the obtained experimental values of DRC of were dichotomized using the median DRC levels. With a median DRC of 4.3%, this study categorized DRC a dichotomous variable: “low” was <4.3%; “high” was ≥4.3%.

### 4.5. Hormone Receptor Status

Pathology reports from all cases were reviewed to confirm the diagnosis, tumor grade and size, presence/absence of axillary lymph node metastasis, and ER, PR, and HER2 status, as previously described [13,18]. Receptor status results were provided by 10 private Puerto Rican laboratories, following ASCO (American Society of Clinical Oncology, Alexandria, VA, USA) and CAP (College of American Pathologists, Northfield, IL, USA) guidelines for those immunohistochemistry (IHC) methods. [36,37] ER and PR results included the percentage of positive-staining cells, the intensity of staining (weak, moderate, or strong), and an interpretation. “Receptor positive” meant ≥1% of tumor cells stained positive for ER/PR [37]. HER2 results were reported as 0, 1+, 2+, or 3+. For this study, any 1+ or 2+ result was considered equivocal and was followed up with FISH (Fluorescence In Situ Hybridization) to so we could categorize HER2 as a dichotomous variable (HER2+ = all 3+ results; HER2− = 2+ to 0 results).

### 4.6. Classification of Tumors Based on IHC Receptor Status Information

Breast cancer subtypes have been defined by others [9,38,39]. Briefly, we used the data collected from the pathology reports on three IHC markers (ER, PR, and HER2) to classify tumors into four groups. Luminal A tumors were ER+, PR+/−, and HER2−. Luminal B tumors differed only by being HER2+. HER2-positive tumors were ER−, PR−, HER2+. Triple-negative tumors were ER−, PR−, HER2.

### 4.7. Statistical Analysis

Descriptive statistics were used to evaluate categorical data as percentages and continuous variables in terms of mean/standard deviation. Chi-square probability distribution was used to assess the statistical relationship between BC subtypes and the following characteristics: DRC, age, onset of menarche, parity and menopause status, BMI, lactation history, alcohol intake, smoking habits, contraceptive use, vitamin intake (multivitamin/calcium), and hormone replacement therapy (HRT) use. To assess the magnitude of the association between DRC and BC subtype, controlling for potential confounders, the following multinomial logistic regression model was used

$$log\frac{P_{k}}{P_{0}}=\beta_{0k}+\beta_{DRC}^{k}+\sum^{\text{​}}\beta_{j}X_{j}$$

where *P_k_* indicates the prevalence of the *k*th-category of BC subtype, *P*_0_ indicates the prevalence of the control group under the DRC comparison (<4.3 vs. ≥4.3), $\beta_{DRC}^{k}$ is the coefficient associated to DRC, *X_j_* indicates the potential confounders, and *β_j_* is the coefficient associated with *X_j_*. Crude and adjusted odds ratios (OR) were estimated with 95% confidence levels from this model. Statistical analyses were performed using Stata v14 (Stata Corp, College Station, TX, USA).

## 5. Conclusions

In general, our findings support what is known about the biology of molecular subtypes of BC. However, the ORs in terms of DRC values do not always match with the basic biology of BC. For example, the lowest OR (2.5) corresponded to Luminal B which is a more aggressive BC than Luminal A (OR = 5.4). This suggests that in terms of DNA repair, it might be important (future direction) to look at other pathways in addition to NER. It is possible that double-strand DNA breaks repaired by homologous and non-homologous end joining might also us to distinguish differences in repair between Luminal A and Luminal B.

This study has some limitations. Our study was based only on the NER pathway and we were not able to measure other pathways. We are now standardizing technology in order to be able to obtain a more comprehensive view of the dysregulation of DNA repair in BC. The HCR assay used is very costly and not easily amenable to large scale population studies such as this one. This assay depends on having viable living cells (lymphocytes) from participants (requires a blood sample) versus genetic tests that can be done with DNA isolated from paraffin embedded tumor samples (no need for live cells or draw blood). However, our study provides the first report evidence of the significant phenotypic variability among the four principal molecular subtypes of BC and provides a hypothesis based on DRC levels for the poor prognosis of TNBC. It suggests that significant phenotypic variability in terms of DRC exists amongst the four molecular subtypes studied.

## Figures and Tables

**Table 1:** Socio-demographic and risk factor distribution by molecular breast cancer subtype.

Socio-demographic Characteristics	Controls *n* (%)	Luminal A *n* (%)	Luminal B *n* (%)	HER2+ *n* (%)	TN *n* (%)	*p*-Value ^a^
*n* = 949	682 (71.9)	157 (16.5)	41 (4.3)	22 (2.3)	47 (4.9)	
**DRC**						<0.001
<4.3	271 (39.7)	122 (77.7)	26 (63.4)	17 (77.3)	39 (83.0)	
≥4.3	411 (60.3)	35 (22.3)	15 (36.6)	5 (22.7)	8 (17.0)	
**Family History of BC**						0.082
No	457 (67.0)	118 (75.2)	33 (80.5)	18 (81.8)	32 (68.1)	
Yes	225 (33.0)	39 (24.8)	8 (19.5)	4 (18.2)	15 (31.9)	
**Age at Diagnosis**						0.040
<45	193 (28.3)	26 (16.6)	6 (14.6)	3 (13.6)	9 (19.1)	
45–55	228 (33.4)	46 (29.3)	15 (36.6)	6 (27.3)	16 (34.0)	
56–65	142 (20.8)	43 (27.4)	15 (36.6)	7 (31.8)	10 (21.3)	
66–75	95 (13.9)	33 (21.0)	4 (9.8)	5 (22.7)	9 (19.1)	
>75	24 (3.5)	9 (5.7)	1 (2.4)	1 (4.6)	3 (6.4)	
Median age ^b^	51 (43, 61)	58 (47, 66)	55 (50, 62)	54 (46, 66)	60 (52, 69)	
**Menopausal Status**						0.004
No	258 (37.8)	39 (25.0)	8 (19.5)	6 (27.3)	13 (27.7)	
Yes	424 (62.2)	117 (75.0)	33 (80.5)	16 (72.7)	34 (72.3)	
Not specified	0	1	0	0	0	
**Age at Menarche**						>0.1
<13	292 (42.9)	74 (48.1)	19 (46.3)	13 (59.1)	22 (46.8)	
≥13	389 (57.1)	80 (51.9)	22 (53.7)	9 (40.9)	25 (53.2)	
Not specified	1	3	0	0	0	
**BMI**						>0.1
<25	236 (34.6)	43 (27.4)	14 (34.2)	10 (45.5)	13 (27.7)	
25–30	258 (37.8)	52 (33.1)	18 (43.9)	5 (22.7)	20 (42.6)	
>30	188 (27.6)	62 (39.5)	9 (21.9)	7 (31.8)	14 (29.8)	
**Alcohol Intake**						0.033
Never	555 (82.6)	138 (89.0)	39 (95.1)	17 (77.3)	43 (91.5)	
Ever	117 (17.4)	17 (11.0)	2 (4.9)	5 (22.7)	4 (8.5)	
Not specified	10	2	0	0	0	
**Smoking Habit**						>0.1
Never	614 (91.1)	137 (87.8)	39 (95.1)	20 (90.9)	43 (91.5)	
Ever	60 (8.9)	19 (12.2)	2 (4.9)	2 (9.1)	4 (8.5)	
Not specified	8	1	0	0	0	
**Oral Contraceptives (Pre-Menopausal Women Only)**						0.054
Never	303 (45.2)	89 (58.2)	22 (53.7)	12 (54.5)	23 (48.9)	
Ever	367 (54.8)	64 (41.8)	19 (46.3)	10 (45.5)	24 (51.1)	
Not specified	12	4	0	0	0	
**Vitamin Intake (Multivitamin or Calcium)**						<0.001
No	333 (48.8)	96 (61.1)	28 (68.3)	14 (63.6)	37 (78.7)	
Yes	349 (51.2)	61 (38.9)	13 (31.7)	8 (36.4)	10 (21.3 )	
**HRT (Post-Menopausal Women Only)**						0.009
Never	205 (48.3)	68 (58.1)	22 (66.7)	12 (75.0)	23 (67.6)	
Ever	219 (51.7)	49 (41.9)	11 (33.3)	4 (25.0)	11 (32.4)	

TN: triple negative; DRC: DNA repair capacity; HRT: hormone replacement therapy; SEM: standard error of the mean; ^a^ *p*-values were computed without missing values (“not specified” information); ^b^ between parentheses percentile 25, and percentile 75.

**Table 2:** Pathological characteristics of tumors by breast cancer molecular subtype.

Pathological Characteristics	Luminal A *n* (%)	Luminal B *n* (%)	HER2+ *n* (%)	TN *n* (%)	*p*-Value ^a^
**Type of Breast Cancer**					>0.1
Carcinoma in situ	7 (4.6)	3 (7.3)	4 (18.2)	1 (2.2)	
Invasive	130 (85.0)	34 (82.9)	17 (77.3)	42 (93.3)	
Mixed invasive	16 (10.4)	4 (9.8)	1 (4.5)	2 (4.4)	
Not specified	2	0	0	2	
**Grade**					<0.001
I	28 (20.3)	2 (5.0)	0 (0.0)	0 (0.0)	
II	81 (58.7)	22 (55.0)	9 (42.9)	14 (31.8)	
III	29 (21.0)	16 (40.0)	12 (57.1)	30 (68.2)	
Not specified	19	1	1	3	

**^a^** *p*-values were computed without missing values. Ductal and lobular tumors were included into each category of carcinoma in situ and invasive BC. Mixed invasive refers to ductal and lobular components within the same tumor.

**Table 3:** Multinomial odds ratio regression analysis for association between DNA repair capacity and breast cancer subtype.

Outcome	DRC<4.3%	DRC ≥ 4.3% (Reference)	Crude OR (95% CI)	Adjusted OR (95% CI) ^a^
Controls (reference)	271	411	1.0	1.0
Luminal A	122	35	5.3 (3.5, 7.9)	5.4 (3.5, 2.8)
Luminal B	26	15	2.6 (1.4, 5.1)	2.5 (1.3, 4.9)
HER2+	17	5	5.2 (1.9, 14.4)	5.2 (1.9, 14.3)
Triple-negative	39	8	7.4 (3.4, 16.1)	7.2 (3.3, 15.7)
TOTALS	475	474		

^a^ Fully adjusted OR: model adjusted for age, menopause status and multivitamin and/or calcium consumption. No significant interaction terms were found in this model using the likelihood ratio test (*p* > 0.05). (*n* = 949).

## Abbreviations

BC	Breast cancer
DRC	DNA repair capacity
NER	Nucleotide excision repair
IHC	Immunohistochemistry
TNBC	Triple-negative breast cancer
BMI	Body mass index
HRT	Hormone replacement therapy
TN	Triple-negative
SEM	Standard error of the mean
OR	Odds ratio
